# Platform shift in mental health support among undergraduates: from campus counselors to GenAI-based consultation​

**DOI:** 10.3389/fpsyt.2026.1827394

**Published:** 2026-06-02

**Authors:** Lin Wang, Yuwei Zang, Yunfeng Xing

**Affiliations:** 1 Department of Student Affairs, Shandong Second Medical University, Weifang, China; 2 School of Agricultural Economics and Rural Development, Renmin University of China, Beijing, China

**Keywords:** generative artificial intelligence, help-seeking behavior, mental health, platform shift, undergraduates

## Abstract

China is advancing the implementation of the “AI+” initiative and exploring the promotion of AI technology in scenarios such as health management and assisted diagnosis. Against this backdrop, universities are accelerating the adoption of GenAI virtual counseling tools to address the growing psychological issues among undergraduates, including depression, anxiety, and academic stress. However, research on why students are shifting from traditional in-person psychological consultations to GenAI consultations remains limited. Therefore, this study focuses on the student population, collecting data through online questionnaires and employing the PLS-SEM method to systematically analyze the key factors and underlying mechanisms influencing students’ willingness to transition from campus psychologists to GenAI virtual doctors. The findings reveal that privacy concerns, social anxiety, and stigma surrounding mental illness significantly enhance students’ inclination toward AI consultations. The comfort level of interacting with AI and the accessibility of AI services play mediating roles. Additionally, perceived AI information quality significantly moderates the pathways through which privacy concerns, social anxiety, and stigma influence the willingness to switch. This study provides a theoretical foundation and practical reference for universities to develop intelligent psychological support systems that align with students’ actual needs.

## 1 Introduction

China’s national “Artificial Intelligence Plus” (AI+) initiative is accelerating the integration of generative artificial intelligence (GenAI) into critical sectors including healthcare management and clinical decision support. Against this policy backdrop, universities are increasingly deploying GenAI-powered virtual counseling tools to address escalating mental health challenges among undergraduates—particularly depression, anxiety, and academic stress. These systems offer distinct advantages over traditional face-to-face therapy: ubiquitous accessibility, reduced stigma through anonymous interaction, and instantaneous response capabilities. Consequently, a paradigm shift is emerging as students demonstrate growing preference for AI-mediated digital support over conventional psychological services when experiencing emotional distress. This behavioral transition represents a critical yet underexplored frontier in educational psychology and human-computer interaction research, necessitating rigorous empirical investigation into its underlying mechanisms and implications.

Existing research primarily focuses on college students’ choices regarding psychological help-seeking, revealing two core categories of factors: those that hinder offline help-seeking and those that promote the use of online services. Hindering factors are mainly concentrated on psychosocial variables such as mental illness stigma, shame, social anxiety, and fear of negative evaluation. Mental illness stigma damages individuals’ self-esteem and help-seeking efficacy through internalization processes, making students reluctant to discuss their mental health needs (1). Shame directly suppresses professional help-seeking behaviors (2).Furthermore, individuals with social anxiety, who have significantly lower help-seeking rates compared to those with other psychological disturbances and tend to avoid social situations, including clinic visits (3, 4), further demonstrate reduced willingness to seek offline help. Additionally, privacy concerns have emerged as a critical variable influencing students’ decisions regarding help-seeking modalities. In contrast, the appeal of online and AI-based psychological services stems from the advantages conferred by their technological characteristics: the high accessibility of new technologies, such as virtual reality, is a core attraction (5). The “non-judgmental” nature of chatbots effectively reduces feelings of shame and fear (6). The affordances for empathetic interaction provided by AI can significantly lessen the pressure of self-presentation and the fear of negative evaluation (7). Moreover, the anytime-available communication mode of AI is particularly suitable for individuals who feel stigmatized by or uncomfortable with traditional therapy (8). Adolescent patients with depression even report lower anxiety levels when using chatbots compared to seeing a human therapist (9). Well-designed AI chatbots can also significantly enhance the accessibility and diversity of mental health services (10).

While existing research provides a foundation for understanding the psychological help-seeking behaviors of college students, insufficient attention has been paid to the dynamic process of their transition from traditional offline counseling to generative artificial intelligence (AI) consultation. This study focuses on the college student population. By collecting data through an online questionnaire and employing Partial Least Squares Structural Equation Modeling (PLS-SEM), it systematically analyzes the key factors and their mechanisms influencing students’ shift from campus psychological counselors to generative AI virtual therapists. Specifically, we explore the effects of mental illness stigma, social anxiety, and privacy concerns on college students’ switching intention. Meanwhile, we discuss the underlying mechanism of AI interaction comfort and accessibility. Moreover, this study examines the interactive effect of perceived AI information quality with mental illness stigma, anxiety, and privacy concerns on students’ switching intention.

This study offers three potential contributions. First, it reconceptualizes the role of psychological barriers in shaping the willingness to switch. While previous research has largely viewed mental illness stigma, privacy concerns, and social anxiety as negative factors hindering students from seeking face-to-face help (3, 11, 12), this study finds that, under specific circumstances, these psychological barriers may instead become drivers propelling students toward AI-based consultation. Second, it reveals the mediating mechanism of AI interaction experience between psychological barriers and switching intention. Existing research has demonstrated that psychological barriers inhibit help-seeking behavior (13). By introducing AI interaction comfort and accessibility as mediators, this study illustrates that psychological barriers indirectly influence switching intention primarily through students’ evaluations of their AI interaction experience. This finding responds to the call by Lattie et al. for digital mental health services to prioritize user experience and provides a process-oriented explanation for understanding how students transition to AI support amid psychological distress (5). Finally, the study clarifies the boundary effects of technology perception factors by validating the moderating role of AI information quality. The results indicate that students’ judgments of AI information quality moderate the strength of this transmission pathway. This aligns with the assertion by Sundar et al. regarding the necessity of ensuring information accuracy in AI and helps explain the differential responses among various student subgroups, thereby offering boundary conditions that refine the proposed model (14).

## 2 Hypothesis development

### 2.1 Privacy concerns, social anxiety, and mental illness stigma

IIn the process of psychological counseling, it is essential for therapists to proactively implement privacy protection measures to maintain trust and integrity, ensuring that sensitive information is properly safeguarded and that clients feel secure throughout the therapeutic process (15). Due to concerns about privacy breaches in offline psychological counseling, and because online AI chatbots are perceived to offer relatively secure privacy protection, a growing number of individuals are turning to AI chatbots for psychological support (16). However, research by Soo et al. indicates that privacy is one of the most critical issues in AI chatbots; users’ emotional responses to privacy risks shape their perceptions of uncertainty, which in turn influences their willingness to switch to AI chatbot-based counseling (17). Accordingly, we propose:

H1. Privacy concerns positively influence the intention to switch.

Social anxiety disorder is characterized by a marked and persistent fear of social situations, wherein individuals are constantly concerned about being evaluated, scrutinized, or judged by others. This distress often hinders them from seeking traditional face-to-face counseling (18). Currently, social anxiety is prompting a growing number of individuals to turn to online AI psychological counseling in search of a more comfortable environment for self-disclosure. Research by Nandnawar et al. indicates that individuals with high social anxiety experience less anxiety and tension when interacting with AI chatbots for counseling compared to interacting with humans, as AI chatbots allow users to engage more comfortably in social skills training and psychological adjustment (19). Similarly, Yang et al. found that among individuals receiving counseling from AI chatbots, symptoms of social anxiety showed significant improvement; by providing customized counseling experiences and immediate feedback, AI chatbots help users alleviate emotional distress and cultivate positive emotional states (20). Accordingly, we propose the following hypothesis:

H2. Social anxiety positively influences the intention to switch.

Individuals with mental health issues often face discrimination or negative evaluations from others due to mental illness stigma, which not only hinders their willingness to seek help but also exacerbates symptoms and diminishes personal well-being (21). For college students in particular, the fear of being judged and concerns about the potential impact on their academic standing and future prospects often lead them to conceal their problems and engage in self-isolation, thereby worsening their condition (22). However, due to the easy accessibility and destigmatizing characteristics of AI chatbots, a growing number of individuals are opting for online AI psychological counseling to avoid the risk of judgment associated with seeking offline professional help (23). Research by Miles et al. indicates that individuals with higher levels of internalized mental illness stigma actually hold more positive attitudes toward AI chatbot-based psychological counseling, as they experience a greater sense of privacy and reduced fear of judgment when interacting with AI chatbots compared to human therapists (24). Accordingly, we propose the following hypothesis:

H3. Mental illness stigma positively influences the intention to switch.

### 2.2 AI interaction comfort and AI accessibility

Individuals’ intention to use AI chatbots is driven by three major factors: privacy concerns, social anxiety, and mental illness stigma, with AI interaction comfort serving as a key mediating mechanism. Regarding privacy concerns, users’ worries about data leakage may hinder their use of AI chatbots. However, when AI chatbots establish a sense of privacy security through technological means, individuals may instead prefer AI chatbots over human counselors (25), as they perceive machine interaction to offer greater confidentiality compared to traditional counseling, reducing the likelihood of content disclosure (26). Individuals with social anxiety traits derive significant comfort benefits from interacting with AI chatbots; the communication environment, which eliminates the need to face others’ gaze, substantially reduces their real-life anxiety, thereby creating a comfortable counseling setting (27). The fear of judgment induced by mental illness stigma leads many patients to avoid traditional therapy and instead prefer AI chatbots, as these tools do not disclose privacy and do not stigmatize individuals with mental health conditions (28). Aktan et al. suggest that individuals with a stronger fear of stigmatization are more inclined to choose AI chatbots, which offer higher levels of interaction comfort (29). Accordingly, we propose:

H4. AI interaction comfort mediates the relationship between privacy concerns and switching intention.

H5. AI interaction comfort mediates the relationship between social anxiety and switching intention.

H6. AI interaction comfort mediates the relationship between mental illness stigma and switching intention.

In addition to AI interaction comfort, AI service accessibility constitutes another core mediating pathway. Privacy concerns, social anxiety, and mental illness stigma enhance users’ perceived value of AI accessibility, thereby increasing their intention to use AI chatbots for online psychological counseling (30). Users who are concerned about privacy breaches in offline counseling tend to choose AI chatbots for online consultation, as these tools enable round-the-clock, anonymous remote communication that protects personal privacy (31). Khan et al. indicate that AI chatbots alleviate the pressure associated with face-to-face counseling for individuals with social anxiety, allowing them to seek psychological support without engaging in real-time social interaction (27). On the other hand, the mediating effect of AI accessibility is also manifested at the psychological level. Research by Miqdadi suggests that individuals who fear mental illness stigma hesitate to seek counseling in offline settings, but AI chatbots provide a space that requires no appointment and eliminates exposure to others’ gaze, enabling them to discuss sensitive topics comfortably (32). This form of accessibility, characterized by low psychological burden, meets the needs of a large population requiring discreet mental health support, positioning AI chatbots as a significant counseling alternative that addresses appointment limitations, specialist delays, and stigmatization concerns (33). Accordingly, we propose:

H7. AI accessibility mediates the relationship between privacy concerns and switching intention.

H8. AI accessibility mediates the relationship between social anxiety and switching intention.

H9. AI accessibility mediates the relationship between mental illness stigma and switching intention.

### 2.3 The moderating effect of perceived AI information quality

Users’ trust in the information quality of AI chatbots stems from their perception of the accuracy, reliability, and unbiased nature of the information provided. This trust evokes positive emotional responses and enhances the acceptance of and satisfaction with AI chatbots (34). Individuals with higher levels of mental illness stigma and privacy concerns tend to trust the information quality of AI chatbots, which positively influences their decision to choose AI chatbots for online psychological counseling (35). However, research by Platt et al. suggests that if the information quality provided by AI chatbots cannot be guaranteed, potentially leading to misdiagnosis or inappropriate advice—it may exacerbate users’ fears regarding AI chatbot therapy, thereby hindering their willingness to engage in online counseling (36). Therefore, perceived AI information quality plays a critical moderating role in the relationships between privacy concerns, social anxiety, mental illness stigma, and users’ intention to switch to online psychological counseling. It may either strengthen the push effect of these factors toward switching or weaken individuals’ willingness to use such services due to concerns about information unreliability (37). Based on this, we propose:

H10a. Perceived AI information quality significantly moderates the relationship between privacy concerns and switching intention through direct or indirect pathways.

H10b. Perceived AI information quality significantly moderates the relationship between social anxiety and switching intention through direct or indirect pathways.

H10c. Perceived AI information quality significantly moderates the relationship between mental illness stigma and switching intention through direct or indirect pathways.

### 2.4 Research framework

**Figure 1:**
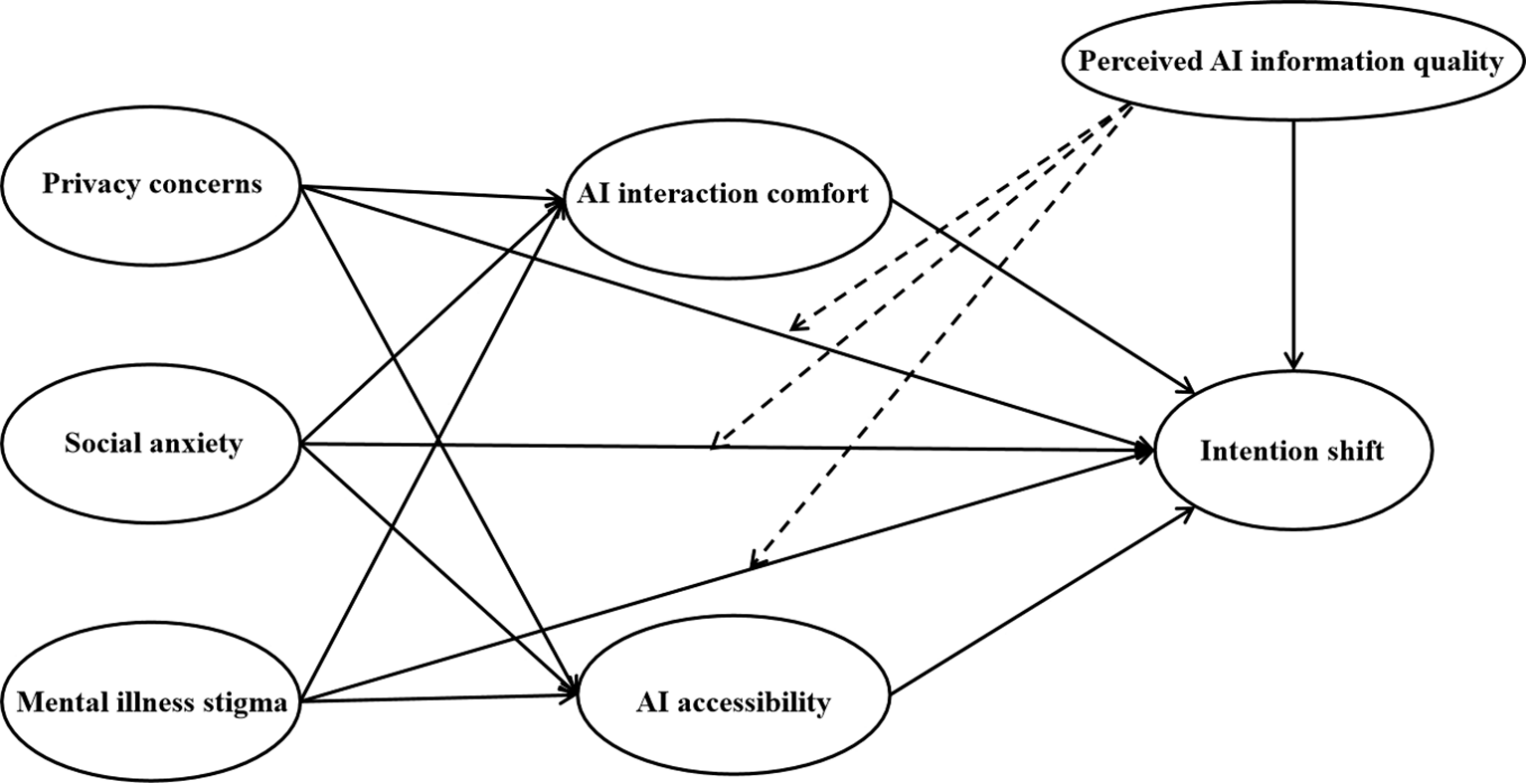
The research framework.

Based on the above analysis, we first introduce privacy concerns, social anxiety, and mental illness stigma as core constructs to investigate the mechanisms through which AI interaction comfort and AI accessibility influence switching intention. Specifically, we focus on analyzing whether there are differences in the effects of privacy concerns, social anxiety, and mental illness stigma on switching intention among Chinese university students. Furthermore, we delve into how privacy concerns, social anxiety, and mental illness stigma influence switching intention, aiming to reveal the underlying mechanisms and potential differences in their pathways of influence. At the same time, considering the complexity of consumer decision-making, we also examine the interactive effect of perceived AI information quality on switching intention. Based on the above analysis, we construct the theoretical framework as shown in **Figure 1**.

## 3 Data and methodology

### 3.1 Sample and data collection

This survey was conducted from January to February 2026, with all respondents being currently enrolled undergraduate or postgraduate students. This particular group was selected as the research subjects based on the following considerations: First, university students are in a critical period of psychological development and face multiple pressures from academics, employment, and interpersonal relationships, making them a significant demand group for mental health services. Simultaneously, they demonstrate high acceptance and frequent usage of new technologies such as AI, rendering them an ideal sample for observing the transition from traditional offline counseling to AI-based consultation services. Furthermore, as this group constitutes the main demographic composition of the university population, encompassing individuals spanning different ages, educational levels, and academic disciplines, it provides us with a representative sample.

The research team distributed the questionnaire link through WeChat groups and invited 70 participants to complete the preliminary survey. We required participants to fill out the questionnaire and evaluate the measurement items in terms of semantic coherence, logical consistency, comprehensibility, and relevance. Meanwhile, we also collected feedback from the participants. Based on the received feedback, we further revised measurement items that presented issues such as ambiguity and lack of coherence, ensuring that the questionnaire content was easy to understand and logically clear. In the formal research phase, we selected Questionnaire Star (https://www.wjx.cn/) as the questionnaire distribution platform. Each respondent received a unique survey link and was permitted to submit only once, thereby preventing duplicate responses. Additionally, we implemented a questionnaire quality screening mechanism, which included requiring participants to provide unique IP addresses and ensuring completion within 8–15 minutes. We also eliminated questionnaires containing a substantial number of missing values, as well as those in which participants provided identical answers to five or more consecutive items. Ultimately, after excluding invalid responses, a total of 904 valid questionnaires were obtained.

The sample characteristics are detailed in **Table 1**. The gender ratio was roughly balanced, with male respondents accounting for 41.48% and female respondents accounting for 58.52%. Of all respondents, 89.27% were aged between 18 and 22 years. Over 77.6% of respondents held a bachelor’s degree. Regarding residence, 49.56% of respondents lived in towns or cities, while 50.44% lived in villages. In terms of academic majors, engineering and science constituted the largest proportions, at 60.18% and 26.22%, respectively. Among the respondents, 41.48% reported frequent use (1–2 times per week) of AI chat tools such as Deepseek, Doubao, and Yuanbao. However, only 10.18% of respondents would exclusively accept online AI counseling, while 54.44% indicated they would accept a mixed approach combining both offline and online AI counseling. Furthermore, 37.39% of respondents had used AI chat tools to seek mental health support.

**Table 1:** Demographic profile of respondents.

Characteristic	Demographic		Frequency	Percentage (%)
Gender	Male		375	41.48
	Female		529	58.52
Age(years)	<18		16	1.77
	18-22		807	89.27
	>22		81	8.96
place of residence	Village		456	50.44
	Town/City		448	49.56
Education level	Undergraduate students	1 st year	144	16.72
		2nd year	140	16.26
		3rd year	126	14.63
		4th year	444	51.57
		Beyond 4th year	7	0.81
	Postgraduate students	1 st year	14	32.56
		2nd year	11	25.58
		3rd year	15	34.88
		Beyond 3rd year	3	6.98
academic major	Natural Sciences		237	26.22
	Engineering		544	60.18
	Management Science		5	0.55
	Agriculture		3	0.33
	Law		3	0.33
	Medicine		80	8.85
	Other		32	3.54
Frequency of using AI chatbots such as DeepSeek, Doubao, Yuanbao, etc.	Never		42	4.65
	Rarely (less than once per month)		64	7.08
	Occasionally (1–3 times per month)		244	26.99
	Frequently (1–2 times per week)		375	41.48
	Very frequently (3 or more times per week)		179	19.8
What do you believe is the most effective mode ofmental health support?	In-person counseling		320	35.4
	AI-driven Counseling		92	10.18
	In-person and AI-driven hybrid counseling		492	54.42
Have you ever used Al-driven mental healthsupport before?	Yes		338	37.39
	No		566	62.61

### 3.2 Measurement

The questionnaire comprised two sections. The first section was used to collect respondents’ demographic characteristics, while the second section was designed to measure privacy concerns, social anxiety, mental illness stigma, AI interaction comfort, AI accessibility, perceived AI information quality, switching intention, and other constructs within the model.

The items in this study were derived from original established scales and modified accordingly. Items for privacy concerns were adapted from Liang et al. (38). Social anxiety was measured based on the scale developed by Heidenreich et al. (39). Mental illness stigma was assessed following the scale established by King et al. (40). AI interaction comfort was adapted from research by Bolpagni et al. (41). Items for AI accessibility and switching intention were developed based on the study by Qiu et al. (42). Perceived AI information quality items were adapted from the relevant research by Almulla et al. (43). All construct items were evaluated using a five-point Likert scale, ranging from “1” (strongly disagree) to “5” (strongly agree).

### 3.3 Analytical technique

The primary technique employed for data analysis was Partial Least Squares Structural Equation Modeling (PLS-SEM). The main reasons for utilizing PLS-SEM are as follows. First, it is suitable for complex models, including those involving multivariate relationships with moderation or mediation effects (44, 45), thus making it appropriate for this study, which encompasses eight constructs. Second, this method does not require the data to strictly adhere to a normal distribution, as its algorithm can transform non-multivariate normally distributed data (46, 47). Third, PLS-SEM possesses high statistical power and is applicable to both exploratory and confirmatory research (48), providing robust explanatory power for this study, which introduces new variables based on traditional theories. Fourth, PLS-SEM offers greater flexibility by addressing factor indeterminacy and avoiding inadmissible solutions (49, 50). Furthermore, the sample size of this study exceeds the threshold of 205 samples required for PLS-SEM (51). Therefore, we applied SmartPLS 4.0 to test the model and employed the bootstrap resampling method for statistical significance testing, with the number of bootstrap sample.

## 4 Data analysis and results

### 4.1 Common method variance and descriptive statistics

We conducted Harman’s single-factor test to examine common method variance (CMV) (52). The results showed that the first factor accounted for 40.412% of the total variance in the factor analysis, which is substantially below the 50% threshold (53). Additionally, **Table 2** reveals no significant correlations. These findings suggest that CMV does not pose a threat to this study. Descriptive statistics of the sample indicate that there were no significant differences among respondents’ privacy concerns (mean = 2.761, SD = 1.221), social anxiety (mean = 3.402, SD = 1.196), and mental illness stigma (mean = 3.411, SD = 1.322). Furthermore, we conducted paired-sample t-tests and found that respondents’ ratings of AI interaction comfort (mean = 3.131, SD = 0.751) and AI accessibility (mean = 3.088, SD = 0.806) showed statistically significant differences. Meanwhile, perceived AI information quality (mean = 3.380, SD = 1.533) and switching intention (mean = 3.008, SD = 0.525) also demonstrated statistically significant differences.

**Table 2:** Reliability and validity tests of the constructs.

Construct	Inner VIF	Outer VIF	Items	Standard loading	Cronbach’s α	CR	AVE
Privacy concerns	1.271	3.292	I’m concerned that the services or institutions I use may not adequately protect my personal information.	0.933	0.935	0.958	0.884
		4.744	When sharing personal information with others or platforms, I am always worried that it may compromise my privacy.	0.947			
		4.634	I worry that my personal information may be leaked by others.	0.941			
Social anxiety-	2.702	3.355	I feel nervous if I have to talk about myself or my feelings.	0.906	0.945	0.961	0.859
		4.464	I have difficulty making eye contact with others.	0.935			
		4.408	I feel anxious when speaking in front of others.	0.931			
		4.677	I feel tense if someone is looking at me.	0.935			
Mental illness stigma	2.841	4.555	I worry that if I tell others I’m seeing a mental health professional, they might not accept me.	0.948	0.935	0.959	0.886
		3.408	I’m afraid that if people find out about my mental health issues, they might discriminate against me.	0.930			
		4.549	I’m concerned that if others know I’m attending counseling, it could damage my relationships.	0.946			
AI interaction comfort	2.626	2.453	I feel that the AI can understand what I say or the questions I ask.	0.904	0.862	0.916	0.784
		2.057	I find the AI’s responses to be clear, concise, and easy to understand.	0.868			
		2.188	The AI makes me feel heard and understood.	0.883			
AI accessibility	2.522	2.513	I can use AI platforms to search for mental health information whenever and wherever.	0.906	0.877	0.924	0.802
		2.313	I find it highly convenient to use AI platforms for searching mental health information.	0.890			
		2.354	I can quickly and easily access the mental health information I need through AI platforms.	0.891			
Intention shift	—	3.718	I am considering switching from the on-campus clinic to an AI platform.	0.931	0.913	0.945	0.852
		2.531	I plan to shift from the on-campus clinic platform to using an AI platform.	0.898			
		4.005	When the AI platform meets the necessary standards, I will replace the on-campus clinic with it.	0.940			
Perceived AI information quality	1.124	1.489	I trust the information provided by the AI to be accurate and reliable.	0.807	0.761	0.862	0.676
		1.709	The AI provides high-quality information relevant to my consultation.	0.832			
		1.506	I trust the information offered by the AI during my counseling process.	0.828			

(1) CR is short for Composite Reliability; (2) AVE is short for Average Variance Extracted.

### 4.2 The measurement model

Cronbach’s alpha and composite reliability (CR) were employed to assess the construct reliability of the scale. As shown in **Table 2**, both Cronbach’s alpha and composite reliability values exceeded the recommended threshold of 0.7, indicating that the scale possesses good internal consistency (54).

Convergent validity, which assesses whether measurement items adequately reflect the same construct, was evaluated using Average Variance Extracted (AVE) and standardized factor loadings (55). The results showed that all item loadings exceeded 0.8, meeting the basic requirement. Furthermore, all AVE values were above 0.6, indicating adequate convergent validity. Discriminant validity was measured using the Heterotrait-Monotrait ratio (HTMT) and the Fornell-Larcker criterion. As shown in **Table 3**, the square root of the AVE for each construct exceeded its correlations with other constructs (56). **Table 4** indicates that all HTMT ratios for construct combinations were below 0.90. Therefore, all constructs demonstrated good discriminant validity (57, 58).

**Table 3:** Correlations and square roots of AVEs (Fornell-Larcker criterion).

Construct	PC	SA	MIS	AIC	AA	IS	PAIQ
PC	0.940						
SA	-0.247	0.927					
MIS	-0.324	0.783	0.941				
AIC	0.213	0.275	0.290	0.885			
AA	0.189	0.262	0.266	0.774	0.896		
IS	0.014	0.650	0.691	0.793	0.730	0.923	
PAIQ	-0.075	0.329	0.279	0.102	0.082	0.246	0.822

PC, Privacy concerns; SA, Social anxiety; MIS, Mental illness stigma; AIC, AI interaction comfort; AA, AI accessibility; IS, Intention shift; PAIQ, Perceived AI information quality.

**Table 4:** Heterotrait-Monotrait ratio (HTMT) and confidence interval.

Construct	PC	SA	MIS	AIC	AA	IS
SA	0.265					
MIS	0.349	0.833				
AIC	0.238	0.303	0.322			
AA	0.207	0.287	0.294	0.890		
IS	0.068	0.701	0.749	0.892	0.815	
PAIQ	0.088	0.385	0.327	0.124	0.100	0.294

PC, Privacy concerns; SA, Social anxiety; MIS, Mental illness stigma; AIC, AI interaction comfort; AA, AI accessibility; IS, Intention shift; PAIQ, Perceived AI information quality.

### 4.3 Path relationship evaluations

**Figure 2:**
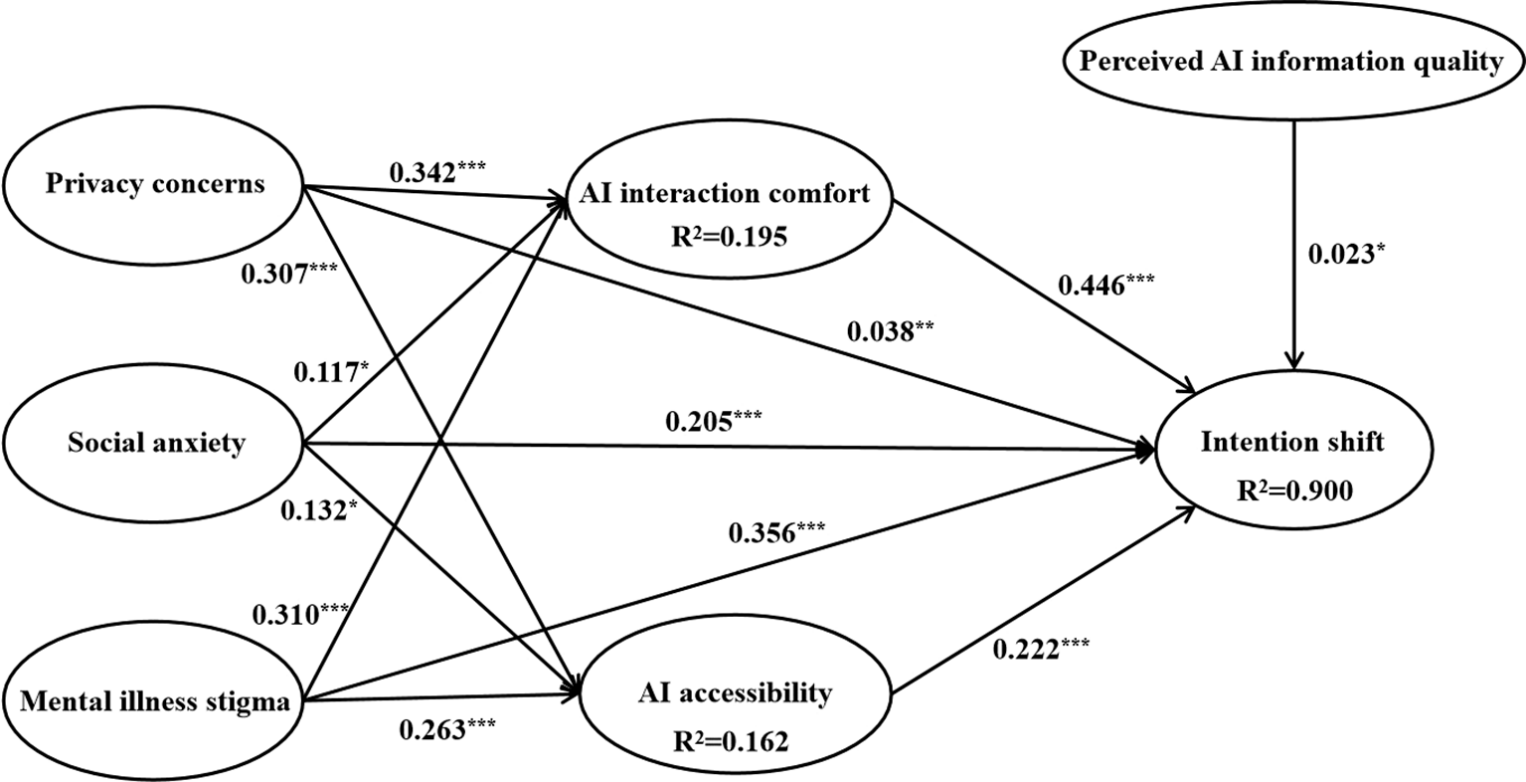
Results of the structural model. *p < 0.05, **p < 0.01, ***p < 0.001.

The results are presented in **Figure 2**. As hypothesized, AI interaction comfort (β = 0.446, p < 0.001) and AI accessibility (β = 0.222, p < 0.001) had positive effects on switching intention. Furthermore, privacy concerns (β = 0.342, p < 0.001), social anxiety (β = 0.117, p < 0.05), and mental illness stigma (β = 0.310, p < 0.001) positively influenced AI interaction comfort. Privacy concerns (β = 0.307, p < 0.001), social anxiety (β = 0.132, p < 0.05), and mental illness stigma (β = 0.263, p < 0.001) positively influenced AI accessibility. Perceived AI information quality (β = 0.023, p < 0.05) had a positive effect on switching intention. Additionally, privacy concerns (β = 0.038, p < 0.01), social anxiety (β = 0.205, p < 0.001), and mental illness stigma (β = 0.356, p < 0.001) positively influenced switching intention directly.

### 4.4 The mediating role of AI interaction comfort and AI accessibility

We employed the bootstrapping method to test the mediating effects. The significance of indirect effects was assessed using 95% confidence intervals and t-values (59). **Table 5** presents the specific indirect effects, total indirect effects, direct effects, and total effects of privacy concerns, social anxiety, and mental illness stigma on switching intention. Accordingly, both AI interaction comfort and AI accessibility serve as significant mediators in the relationships between privacy concerns, social anxiety, mental illness stigma, and switching intention. Furthermore, the specific mediating effect of AI interaction comfort on the relationship between privacy concerns and switching intention (β = 0.153, p < 0.001) was larger than the mediating effect of AI accessibility (β = 0.068, p < 0.001). In contrast, the specific mediating effect of AI interaction comfort (β = 0.052, p < 0.05) was higher than that of AI accessibility (β = 0.029, p < 0.05) in the relationship between social anxiety and switching intention. Additionally, the specific mediating effect of AI interaction comfort on the relationship between mental illness stigma and switching intention (β = 0.138, p < 0.001) was greater than the mediating effect of AI accessibility (β = 0.058, p < 0.001). Moreover, the total effect of mental illness stigma (β = 0.552, p < 0.001) was more significant than that of privacy concerns (β = 0.258, p < 0.001) and social anxiety (β = 0.287, p < 0.001).

**Table 5:** The results of the mediating effect.

Hypotheses and paths		PC→AIC→IS	PC→AA→IS	SA→AIC→IS	SA→AA→IS	MIS→AIC→IS	MIS→AA→IS
Specific indirect effects	β	0.153***	0.068**	0.052*	0.029*	0.138***	0.058***
	T-value	8.563	5.439	1.869	2.041	4.948	3.736
	Confidence intervals	[0.120, 0.190]	[0.045, 0.095]	[-0.003, 0.107]	[0.002, 0.059]	[0.086, 0.196]	[0.030 0.092]
Total indirect effects	β	0.221***		0.081**		0.197***	
	T-value	11.192		2.488		5.750	
	Confidence intervals	[0.183, 0.261]		[0.017, 0.144]		[0.131, 0.266]	
Direct effects	β	0.038**		0.205***		0.356***	
	T-value	2.765		9.016		14.130	
	Confidence intervals	[0.015 0.060]		[0.168 0.243]		[0.314, 0.397]	
Total effects	β	0.259***		0.286***		0.553***	
	T-value	13.260		9.070		17.755	
	Confidence intervals	[0.221, 0.297]		[0.224, 0.348]		[0.491, 0.612]	

*p < 0.05. **p < 0.01. ***p < 0.001.

PC, Privacy concerns; SA, Social anxiety; MIS, Mental illness stigma; AIC, AI interaction comfort; AA, AI accessibility; IS, Intention shift.

### 4.5 The moderating role of perceived AI information quality

We added calculated interaction terms to the model based on Chin et al. (60). **Table 6** shows that perceived AI information quality is a significant moderator of the indirect effects on switching intention. Specifically, perceived AI information quality strengthened the relationship between social anxiety and switching intention (β = 0.100, p = 0.001). However, its moderating effects on the relationships between privacy concerns and switching intention (β = -0.005, p = 0.344) and between mental illness stigma and switching intention (β = -0.016, p = 0.141) were not significant.

**Table 6:** The results of the moderating effect.

Moderator variable	Interacting	Dependent variable	β	p
PAIQ	PAIQ*PC	IS	-0.005	0.344
PAIQ	PAIQ*SA	IS	0.100	0.001
PAIQ	PAIQ*MIS	IS	-0.016	0.141

PC, Privacy concerns; SA, =Social anxiety; MIS, Mental illness stigma; PAIQ, Perceived AI information quality.

### 4.6 Predictive relevance

Cross-validated redundancy and the coefficient of determination (R²) were employed as primary indicators to assess predictive relevance (55). R² serves as the main criterion for evaluating the overall predictive power of the model. Values below 0.3, between 0.3 and 0.6, and above 0.6 represent small, medium, and large predictive power, respectively (52). The R² values for AI interaction comfort and AI accessibility were 0.195 and 0.162, respectively, indicating small predictive power, whereas the R² value for switching intention was 0.900, indicating large predictive power. Moreover, the sample size of this study is 904, which exceeds the threshold of 10 times the number of main variables. As shown in **Table 2**, both the internal and external VIF values are below the threshold of 5. The adjusted R² shows no substantial change compared with the original R². Therefore, there is no overfitting problem in the model. Relative predictive relevance was assessed using the Stone-Geisser method (Q²), obtained through the blindfolding procedure (61, 62). Q² values greater than zero generally indicate that predictive accuracy is considered acceptable and meets the standard criterion (55). Specifically, Q² values between 0.02 and 0.15, 0.15 and 0.35, and above 0.35 indicate small, medium, and large effect sizes, respectively (63). The Q² values for AI interaction comfort and AI accessibility were 0.151 and 0.129, respectively, indicating medium effect sizes. Furthermore, the Q² value for switching intention was 0.762, indicating a large effect size. The results for R² and Q² are presented in **Table 7**.

**Table 7:** Fit indices for the model in the study.

Construct	R^2^	Adjusted R^2^	Q^2^
AIC	0.195	0.192	0.151
AA	0.162	0.160	0.129
IS	0.900	0.899	0.762

AIC, AI interaction comfort; AA, AI accessibility; IS, Intention shift.

## 5 Discussion

This study focuses on university students’ switching intention from offline to online mental health counseling and systematically investigates the direct effects of three key factors—privacy concerns, social anxiety, and mental illness stigma—as well as the mediating roles of AI interaction comfort and AI accessibility and the moderating effect of perceived AI information quality.

First, privacy concerns, social anxiety, and mental illness stigma are all critical factors influencing individuals’ intention to switch to AI-assisted mental health services, yet their effects vary in strength. Specifically, the total effect of mental illness stigma on individuals’ intention to switch to AI services is stronger than that of privacy concerns and social anxiety. Possible reasons lie in the fact that mental illness stigma makes students fear most that their psychological problems will be discovered by classmates, teachers, and family members, as this may lead to discrimination or being labeled negatively (64). In contrast, anonymous AI counseling can fully conceal personal identity and avoid the risk of stigmatization, which explains why its influence is far stronger than that of privacy concerns and social anxiety. In addition, when students perceive lower privacy risks, their trust in AI services increases accordingly (65). Similarly, the online environment relieves the pressure of face-to-face interaction among individuals with social anxiety (66). Therefore, all three factors significantly influence individuals’ willingness to switch to AI-assisted mental health services. Notably, 62.61% of respondents in this study reported that they had never used AI-driven mental health support services, indicating a clear gap between behavioral intention and actual usage behavior. Although students hold positive intentions toward AI mental health tools, the current limitations of generative AI, including insufficient professional competence, poor emotional understanding, as well as ethical and security risks, may hinder the translation of intention into actual behavior.

Second, privacy concerns, social anxiety, and mental illness stigma influence individuals’ intention to switch to AI-assisted mental health services through two pathways: AI interaction comfort and AI accessibility, with the mediating effect of AI interaction comfort being stronger than that of AI accessibility. The immediate responsiveness, personalized interaction capabilities, and role of AI chatbots as complementary tools to traditional offline counseling position them as significant options in the field of mental health support (67). Individuals with social anxiety tend to rely more on their comfort experience during the consultation process when making decisions and are more sensitive to changes in AI interaction comfort (68). The relatively weaker mediating effect of AI accessibility suggests that university students’ core motivation for choosing online counseling stems from emotional needs rather than instrumental needs. This provides new empirical evidence for understanding why people turn to AI psychological counseling from the perspective of emotional needs.

Finally, perceived AI information quality, as a moderating variable, significantly strengthens the pathway between social anxiety and switching intention, but its moderating effects on the relationships involving privacy concerns and mental illness stigma are not significant. Specifically, when users perceive higher AI information quality, the positive driving effect of social anxiety on the intention to switch to AI services is significantly amplified. This moderating effect arises because high-quality information, by enhancing transparency and interpretability, alleviates the fear of interpersonal interaction among individuals with social anxiety, thereby encouraging them to adopt AI services more actively. However, privacy concerns and mental illness stigma are long-term cognitive beliefs shaped by social stereotypes and risk perceptions, which remain unchanged by situational AI information quality. Privacy concerns largely depend on institutional data security regulations, while mental illness stigma is deeply rooted in pervasive social prejudice, rendering both insensitive to the informational characteristics of AI (69). In contrast, social anxiety is a flexible, situationally affected emotion that is highly susceptible to external informational cues (70). Consequently, the moderating effect of perceived AI information quality on the relationships between privacy concerns and switching intention, as well as between mental illness stigma and switching intention, did not reach statistical significance.

## 6 Conclusions, implications, and limitations

### 6.1 Conclusion

Privacy concerns, social anxiety, and mental illness stigma are key factors driving students’ switching to AI-based counseling. Users’ interaction comfort with AI and the accessibility of AI services play significant mediating roles in the pathways influencing their acceptance and usage intentions. Perceived AI information quality significantly moderates the aforementioned relationships: when students perceive the psychological information provided by generative AI as accurate and trustworthy, the positive effects of social anxiety on their comfort with and accessibility to AI use are strengthened, thereby further promoting their help-seeking intentions (71).

### 6.2 Implications

The findings of this study offer significant practical guidance for mental health services in higher education institutions and for the design of AI products.

First, universities should acknowledge the inherent barriers present in traditional psychological counseling services. Inadequate privacy protection, social anxiety, and stigma concerns are underlying factors that constrain service utilization. Although AI tools cannot fully replace human-delivered services, they can serve as low-threshold entry points, effectively mitigating the obstacles students face when seeking psychological help and reducing avoidance behaviors stemming from such concerns (72). This finding suggests that universities and AI enterprises should strengthen collaboration to construct an “online + offline” blended mental health service system. For instance, online counseling could function as a preliminary step to help students alleviate social anxiety before guiding them toward participating in in-depth offline counseling sessions; simultaneously, AI tools could provide personalized recommendations to enhance service precision.

Second, given the strong mediating effect of interaction comfort on university students’ willingness to switch to AI-based psychological support services, universities should establish a dual-spiral design framework integrating emotional and functional elements during system development, positioning emotional adaptability as a core experiential factor. Specifically, this involves employing anthropomorphic interaction technologies to analyze students’ micro-tremors in speech, micro-expression features, and behavioral trajectories in real time, thereby dynamically adjusting response strategies accordingly. For example, by integrating voice emotion recognition and facial expression analysis, AI systems can identify emotional states such as anxiety and depression and switch to empathetic communication strategies accordingly. Simultaneously, culturally adapted expressions should be incorporated through the construction of localized semantic networks and the embedding of traditional cultural imagery to enhance users’ emotional resonance. Although the mediating effect of accessibility is relatively weak, it remains necessary to optimize immediate response mechanisms, streamline usage procedures, and protect student data privacy through encryption technologies to prevent disclosures.

Finally, as a key moderating variable, perceived AI information quality exerts a significant amplifying effect on university students’ willingness to switch to AI-based psychological support services. Universities should pursue a dual-path approach encompassing both content governance and literacy cultivation to optimize information quality and guide rational usage. On one hand, a professional review system for AI psychological content should be established, with rigorous scrutiny regarding scientific accuracy, safety, and ethical considerations, ensuring that output information meets clinical psychology standards and guaranteeing information reliability at the source. On the other hand, targeted education on AI health literacy should be provided to students, focusing on enhancing their ability to discern the credibility of AI-generated content, thereby preventing risks arising from misplaced trust or excessive dependence (71, 73). It is worth noting that improving information quality itself can indirectly alleviate the inhibitory effect of social anxiety on switching intention by enhancing user trust; when students perceive AI information as both professional and empathetic, they will more actively embrace AI services.

### 6.3 Limitations

This study has three limitations. First, the use of cross-sectional data restricts the ability to capture the dynamic process of how switching intention translates into actual usage behavior over time. Future research should adopt longitudinal designs to track the evolutionary trajectory of user behavior, examining whether initial intentions lead to sustained engagement and exploring the determinants of the transition from trial use to long-term adoption. Second, this study focuses on individual psychological factors among students, without incorporating macro-contextual variables such as organizational support, faculty attitudes, or technological infrastructure. Future research should employ multilevel modeling approaches to examine the influence of factors including institutional culture, resource allocation, and technological readiness on the implementation effectiveness of GenAI systems, thereby providing a more comprehensive understanding of the ecological validity of such systems. Finally, the sample for this study was drawn from China, and thus the findings may not be generalizable to other cultural contexts. Future research should conduct cross-cultural comparisons by including samples from diverse cultural backgrounds to test the universality of the proposed model and to explore how cultural dimensions shape the human-machine therapeutic alliance.

## Data Availability

The original contributions presented in the study are included in the article/supplementary material. Further inquiries can be directed to the corresponding author.
